# A novel antibacterial approach of Cecropin-B peptide loaded on chitosan nanoparticles against MDR *Klebsiella pneumoniae* isolates

**DOI:** 10.1007/s00726-023-03356-4

**Published:** 2023-11-15

**Authors:** Hend Okasha, Heba Dahroug, Abdullah E. Gouda, Mohamed Abbas Shemis

**Affiliations:** 1 Biochemistry and Molecular Biology Department, Theodor Bilharz Research Institute, Giza, Egypt; 2 Microbiology Department, Theodor Bilharz Research Institute, Giza, Egypt

**Keywords:** Egypt, Multidrug-resistant, *Klebsiella pneumoniae*, Cationic antimicrobial peptide, Cecropin, Bactericidal, Chitosan, Nanocapsule, PCR, Antibacterial

## Abstract

Egypt has witnessed the emergence of multidrug-resistant (MDR) *Klebsiella pneumoniae*, which has posed a serious healthcare challenge. The proper treatment choice for MDR-KP infections is not well determined which renders the problem more complicated, thus making the control of such infections a serious challenge for healthcare professionals. This study aims to encapsulate the cationic antimicrobial peptide; Cecropin-B (Cec-B), to increase its lifetime, drug targeting, and efficacy and study the antimicrobial effect of free and encapsulated recombinant rCec-B peptide on multidrug-resistant *K. pneumoniae* (MDR-KP) isolates. Fifty isolates were collected from different clinical departments at Theodore Bilharz Research Institute. Minimal inhibitory concentrations (MICs) of rCec-B against MDR-KP isolates were determined by the broth microdilution test. In addition, encapsulation of rCec-B peptide into chitosan nanoparticles and studying its bactericidal effect against MDR-KP isolates were also performed. The relative expression of efflux pump and porin coding genes (*ArcrB*, *TolC*, *mtdK*, and *Ompk35*) was detected by quantitative PCR in treated MDR-KP bacterial isolates compared to untreated isolates. Out of 60 clinical MDR isolates, 50 were MDR-KP. 60% of the isolates were XDR while 40% were MDR. rCec-B were bactericidal on 21 isolates, then these isolates were subjected to treatment using free nanocapsule in addition to the encapsulated peptide. Free capsules showed a mild cytotoxic effect on MDR-KP at the highest concentration. MIC of encapsulated rCec-B was higher than the free peptide. The expression level of genes encoding efflux and porin (*ArcrB*, *TolC*, *mtdK*, and *Ompk35*) was downregulated after treatment with encapsulated rCec-B. These findings indicate that encapsulated rCec-B is a promising candidate with potent antibacterial activities against drug-resistant *K. pneumoniae.*

## Introduction

The emergence of multidrug-resistant (MDR) and extensively drug-resistant (XDR) bacteria, combined with the failure of most current therapeutics in addition to the decline in new antibiotic development, poses a severe threat to global public health. *Klebsiella pneumoniae* is a major drug-resistant pathogen associated with community-acquired (CA) and hospital-acquired (HA) infections. The World Health Organization (WHO) recently published a global priority list of antibiotic-resistant bacteria, where carbapenem-resistant Enterobacteriaceae, including *K. pneumoniae,* was incorporated in the Priority 1 group (Freitas and WHO 2017). Reports of MDR *K. pneumoniae* came from numerous Egyptian governorates, where infections appear to be growing faster than ever, necessitating strict infection control (Sherif et al. 2021; Gamal et al. 2016). Considering the further limitations of antimicrobial options and the high mortality rate associated with these infections, there is an urgency to develop new antimicrobial strategies to cope with these XDR pathogens.

Penicillins, carbapenems, and cephalosporins are only a few of the β-lactams that the β-lactamase-producing *K. pneumoniae* may break down. The main resistance mechanisms of *K. pneumoniae* mainly include the production of β-lactamase, the lack of membrane porin proteins, and the active efflux of antibacterial drugs (Papp-Wallace et al. 2011).

Bacterial efflux pumps carry various drugs out of the cell and provide multidrug resistance. Certain pumps within Gram-negative bacteria form multi-protein structures that traverse the cell membrane. These assemblies consist of an outer membrane protein, a plasma membrane-spanning protein, and a periplasmic protein that connects the two trans-membrane components. The RND-based tripartite efflux pump AcrAB-TolC cycles through three different conformational states when transporting drugs. It is made up of the inner membrane transporter AcrB, the periplasmic membrane fusion protein AcrA, and the outer membrane protein TolC (Wang et al. 2017). Two trimeric porins, OmpK35 and OmpK36, are responsible for antimicrobial drug penetration in *K. pneumoniae,* to reach the periplasm, antimicrobial drugs must first pass through the outer membrane. With β-lactams, which are often hydrophilic and charged, porin channels appear to be the primary path of passage (Sugawara et al. 2016).

The existence of multidrug resistance limits the range of antibiotic options available for conclusive therapeutic interventions. The use of abandoned antibiotics such as polymyxins and drug combinations have been introduced as a solution; however, it remains difficult to determine what combination would be most effective in any given clinical situation (Natan and Banin 2017). This makes alternative approaches to combat infections caused by MDR pathogens urgently needed and even mandatory (Ahmed et al. 2020).

Antimicrobial peptides (AMPs) are promising candidates to overcome the abovementioned drug-resistance crisis. Several characteristics make them potential therapeutic alternatives to antibiotics.The crucial attributes include standardized synthetic procedures, swift antimicrobial efficacy, a wide spectrum of antimicrobial activity, and a diminished likelihood of resistance development (Pandit et al. 1862*).*

Specifically, the use of cationic AMPs (CAMP) is emerging as a promising non-antibiotic therapeutic strategy to overcome resistance as they have shown to be highly effective in killing bacterial strains resistant to conventional antibiotics (Ocampo-Ibáñez et al. 2020). Cecropins were first discovered in Cecropia moth (*Hyalophora cecropia*) pupae (Swithenbank et al. 2020; Zhong et al. 2013; Okasha et al. 2021). The cationic low-molecular-weight hemolymph proteins appear upon the intrusion of bacteria. The majority of cecropins have a helix–hinge–helix structure and are made up of an amphipathic N-terminal region and a hydrophobic C-terminal component (CDC 2011). One of the cecropins’ antibacterial proteins that has received the most research is Cecropin-B. In addition, a variety of lepidopteran, dipteran, and coleopteran insects have chemicals similar to cecropin (Campos et al. 2021). Among the various cecropins, A, B, and D stand out as the three primary cecropins. In terms of antibacterial effectiveness, B exhibits the highest potency against bacteria, establishing the order as B > A > D. Cecropin-B is characterized by a molecular weight of 3.84 kDa (35 amino acids) holding + 7 net positive charge at pH 7.0. Out of 35 amino acids, 17 amino acids show hydrophobicity.

However, clinical applications of these AMPs have been hampered by several problems, such as cytotoxicity to host cells, low stability, and hepatic clearance mechanisms resulting in their poor bioavailability from oral and non-oral mucosal routes and inactivity at physiological salt concentrations (Sarkar et al. 2021). Thus, the above obstacles have to be considered when searching for or developing newly effective AMPs for therapeutic use. So, modification in peptide structure through conjugation or encapsulation will increase their tolerability (Okasha 2023).

Notably, chitosan is gaining importance as an antibacterial agent since bacteria are not reported to develop resistance to it (Ghanbari and Roushani 2018). Chitosan nanoparticles (CSNPs) were initially synthesized in 1994 by Ohya and colleagues (Zuhairah Zainuddin and Abdul Hamid 2021). Many researchers have shown that chitosan nanoparticles and their derivatives have antibacterial properties. Chitosan-based drug formulation has gained attention for its ability to assist as a carrier and an enhancer for the oral delivery of peptides and vaccines. Recently, there has been significant research interest in the application of chitosan as an enzyme inhibitor, mucoadhesive agent, and efflux pump inhibitor. Interaction of positively charged amino groups of chitosan with negatively charged sialic acid groups that exist in mucin extends the residence time between drugs and membranes, therefore enhancing the bioavailability of the drugs. Also, peptide aggregation can be avoided, thus enhancing the absorption of peptide drugs in the intestinal epithelium. (Zuhairah Zainuddin and Abdul Hamid 2021; Muheem et al. 2016).

Chitosan’s antimicrobial properties made it a perfect choice in the delivery of oral antibiotics to eradicate Gram-negative bacteria such as *E. coli* (Goy et al. 2016). This approach not only enhances the bioavailability of antibiotics in the body but also indirectly improves the effectiveness of the drugs in eradicating the infection (Radwan-Pragłowska et al. 2019). Previously, Cec-B was produced in *E. coli* using recombinant DNA technology. The purified rCec-B had much less cytotoxicity on normal human WI-38 cells and the IC_50_ was ≤ 1.469 mg/ml (Okasha and Nasr 2021).

To the best of our knowledge, this is the first study to evaluate the antibacterial activity of the antimicrobial effect of encapsulated rCec-B against MDR/XDR *K. pneumoniae* clinical isolates as well as to elucidate the anti-efflux and porin potential of this peptide against MDR/XDR *K. pneumoniae* biofilms.

We aimed to assess the effect of encapsulated rCec-B on MDR isolates of *Klebsiella pneumoniae* using the microdilution broth method. The impact of these chitosan-loaded NPs with and without Cecropin-B was analyzed by the relative expression of efflux pump and porin resistance genes using real-time PCR.

## Materials and methods

### Materials

Chitosan, low-molecular-weight, deacetylated chitin, poly (D-glucosamine), glacial acetic acid, and sodium tripolyphosphate (TPP) were purchased from Sigma-Aldrich (Germany). Phosphate buffer saline (PBS), sodium hydroxide, and hydrochloric acid were supplied by Loba Chemical (India). Ultrapure water with a resistivity of 18 MU cm was used in all aqueous preparations. Recombinant Cecropin-B (rCec-B) peptide (free peptide) was a final product of a Research project (grant No#28/K) supported by Theodor Bilharz Research Institute (TBRI), Giza, Egypt.

### Ethics approval

The study’s samples were all preserved, and codes rather than patients’ names were used. The study’s protocol was authorized by the institutional review board of TBRI under Federal Wide Assurance (FWA00010609/PT-691), and the work was done in compliance with the Declaration of Helsinki for Experiments in Humans.

### Methods

#### Bacterial strains and growth conditions

Over 1 year, 60 isolates of multidrug-resistant Gram-negative bacteria were collected from the TBRI microbiology clinic, where 50 out of 60 MDR isolates were *K. pneumoniae* which was selected for further study. The isolates were collected from different inpatient and outpatient clinics, and the sample sources were respiratory secretions, urine, and blood. The cultures of isolates were sent to a Microbiology Laboratory at TBRI, in the context of laboratory surveillance, where the bacterial species were confirmed, and the antibiotic susceptibility characterization was performed.

#### Characterization of clinical isolates: resistance profile and identification of resistance genes

Following their isolation, the identification of Gram-negative colonies was primarily done by conventional microbiologic methods. The phenotypical and genotypical characterizations of the resistance of clinical isolates were performed. For phenotypic characterization, the resistance and susceptibility of the bacterial strains were determined by the disc diffusion method. Antibiotic discs included amikacin, aztreonam, amoxicillin/clavulanic acid, cefotaxime, tetracycline, piperacillin/tazobactam, ceftazidime, trimethoprim/sulphamethoxazole, ciprofloxacin, cefaclor, cefepime, imipenem, meropenem, cefoxitin, minocycline, and nitrofurantoin for urine samples only. All discs were purchased from Hi-Media Chemicals Pvt. Ltd., Mumbai, India. The interpretation of inhibition zone diameters was conducted in accordance with the interpretative standards for zone diameters outlined in the guidelines of the Clinical Laboratory Standards Institute, as specified by the Kirby–Bauer method (CATALOG 2018). Multidrug-resistant (MDR) bacteria were considered if the bacterial isolate was non-susceptible to at least one agent in three or more antimicrobial categories (25). The classification of the isolate as extensively drug-resistant (XDR) was assigned when it demonstrated non-susceptibility to at least one agent in all antimicrobial categories except two or fewer. Isolates screened for PCR amplification checked by real-time PCR (qPCR) using specific primers for detection of efflux pump coding genes (*AcrAB*, *TolC*, *mtdK*) and porin coding gene *Ompk35 *was also investigated.

#### Testing integrity of Cec-B peptide using FPLC

To assure the stability of the free peptide, a sample of the rCec-B (100 µg/ml) was preserved in LB media (pH 7) at 37 °C for 24 h to be a negative control of free peptide for testing the peak integrity as a peptide stability test. Buffer exchange in 20 mM Tris pH 8 was performed using Vivaspin-500 with molecular weight cutoff 3 kDa (Sartorius Co). Using HiTrap SP-FF (1ml) cation exchange chromatography column (GE Healthcare Life Sciences) and AKTA Purifier 100 FPLC system (GE Healthcare Life Sciences, Sweden), rCec-B was tested for its integrity. Linear gradient elution was carried out by the volume of 20 column volumes and an increase in ionic strength to the used as elution buffer B (20 mM Tris pH 8 and 1M NaCl) (Duong-Ly and Gabelli 2014).

#### Synthesis of chitosan/Cec-B nanoparticles

Chitosan as a natural polymer and sodium tripolyphosphate (TPP) as a cross-linker were used to prepare the nanoparticles according to a slightly modified ionotropic gelation method. Particles with and without Cec-B were prepared in the same condition. Particles without Cec-B were used as a negative control. Different trials were accomplished to optimize the most accurate conditions for nanoparticle preparation. Then 2 mg/ ml chitosan (CS) in 1% (*v*/*v*) acetic acid (Thomas et al. 2016) and 1mg/ ml of TPP aqueous solution were prepared and filtrated using a 0.45 μm syringe filter (from VWR). The pH level of the chitosan and TPP solutions was adjusted to 5.5 by the addition of NaOH and HCl, respectively. For the experiments with Cec-B encapsulation, the peptide was diluted to a concentration of 50 µg∕ ml with the chitosan solution. A glass burette was adjusted to add the TPP solution to the chitosan solution (at a TPP to chitosan weight ratio of 1:2) dropping wisely with the flow rate of 1.25 mL/min. During the addition, the chitosan solution was stirred vigorously (1000 rpm) using a magnetic stirrer. The solution was mixed for an additional 10 min after all of the TPP was expended. The nanoparticle mixture was incubated at 4 °C for 40 min and then centrifuged at 18,000 rpm for 50 min at 4 °C. The supernatant was collected for calculating the efficiency of entrapment and the nanoparticles pellet was washed three times with double distilled water to remove liberated Cec-B. After three washes, the pellet was liquified (resuspended) in double distilled water (5 μg/100 μL) and stored at −80 °C till freeze-dried. Subsequently, the samples were transferred to the freeze dryer under the standard freeze-drying conditions (pressure 7 bars, the inlet temperature was 96 ºC, and the achieved outlet temperature (65–70 °C) (Degobert and Aydin 2021). All experiments were done at room temperature.

#### Efficiency of entrapment

The percent efficiency of entrapment (%EE) of the Cec-B entrapped or adsorbed onto the chitosan was obtained from the determination of free Cec-B concentration in the supernatant recovered after particle centrifugation (18,000 rpm, 50 min) by absorbance measurement at λmax = 210 nm. These Cec-B quantities were determined using a Multiskan sky spectrophotometer (Thermo Scientific, Germany). The supernatant obtained from chitosan-TPP nanoparticles without Cec-B was utilized as a blank (Sedyakina et al. 2020; Masalova et al. 2013).

Cec-B entrapment efficiency (%) was the percentage of entrapped Cec-B to the total amount of Cec-B added. The %EE was calculated using Eq. 1:

1 $${\text{Efficiency of entrapment = }}\left( {{\text{Cec}} - {\text{ B}}_{0} - {\text{Cec }} - {\text{ B}}_{{\text{f}}} {\text{/Cec }} - {\text{ B}}_{0} } \right)$$

where Cec-B_0_ is the initial amount of Cec-B added for encapsulation and Cec-B_f_ is the amount of non-entrapped Cec-B in the supernatant after centrifugation of the particles, respectively. Also, the loading capacity of Cec-B onto the chitosan particles was determined according to Eq. 2:

2 $${\text{Loading capacity }} = \left( {{\text{Cec - B}}_{0} {-}{\text{ Cec - B}}_{{\text{f}}} } \right)/{\text{NPs wt x 1}}00$$

where NPs wt is the weight of the recovered particles.

#### Characterization of the chitosan/rCec-B nanoparticles

Malvern Zetasizer Nano ZS (Malvern Instruments Ltd., Malvern, Worcestershire, UK) was used to determine the particle size distribution, zeta potential, and polydispersity index (PDI). Typically, 2 mg of chitosan and chitosan/Cec-B NPs were resuspended in 2 mL of 1% acetic acid solution; it was sonicated for 10 min to ensure uniform dispersion. The particle size analysis of the nanoparticles was performed in triplicates at 25 °C, an angle of 90° for the photomultiplier, and a wavelength of 633 nm. The surface charge (zeta potential) of the nanoparticles was determined from the electrophoretic mobility. The zeta potential measurements were carried out at pH 5.5 in triplicates using the 100 μL aqueous dip cell by Zetasizer, Nano ZS (Malvern Instruments Ltd., Malvern, Worcestershire, UK). The samples were diluted to 1: 100 with double distilled water before measuring.

#### FTIR analysis

The Fourier transform infrared (FTIR) spectra of chitosan, chitosan unloaded NPs, and chitosan/Cec-B NPs were obtained with a Bruker Vertex 80 IR spectrometer (Germany) from 4000 to 400 cm^−1^, at a resolution of 4 cm^−1^, and reflective index 2.4.

#### Swelling kinetics of chitosan/Cec-B nanoparticles

The swelling behavior of the cross-linked chitosan NPs was measured by swelling the NPs in PBS of different pHs at room temperature and in deionized water at 4, 25, 37, and 42 °C. Dry nanoparticles, initially weighed (around 20 mg), were submerged in buffer solutions spanning pH 2.5 to 8.5. Additionally, the impact of ionic strength on the swelling ratio was investigated by employing various concentrations of sodium chloride solution (50, 100, 200, 400, and 800 mM). The NPs were withdrawn from the solutions at different time intervals and their wet weight was determined after blowing with a stream of air to remove the surface water and immediately weighing the NPs. The swelling ratio was calculated using the equation

3 $${\text{Sr }}\left( \% \right) \, = \, \left( {\left( {{\text{Ws}} - {\text{Wd}}} \right) \, /{\text{ Wd}}} \right) \, \times { 1}00$$

where Sr is the water absorption (%wt) of the NPs, and Wd and Ws are the weights of the samples in the dry and swollen states, respectively.

#### In vitro drug release of the chitosan/Cec-B nanoparticles

First, 5 mg of chitosan–Cec-B nanoparticles were added to 5 ml 1 × PBS buffer of pHs 2.5, 5.5,7.4, and 8.5, then incubated at 37 °C in an orbital stirring shaker at 100 rpm. An aliquot of supernatant (500 μl) was taken out at time intervals and supplemented with 500 μl fresh 1 × PBS buffer (pH 2.5, 5.5, 7.4, and 8.5) to maintain the total volume of the four tubes. The absorbance of released Cec-B at the various pHs was measured using a Multiskan sky spectrophotometer (Thermo Scientific, Germany), at λmax = 210 nm according to the unique calibration curve.

#### Hemolytic activity of the chitosan/Cec-B nanoparticles

Collected blood in a heparin tube from a healthy donor was used to study the hemolytic effect of free chitosan nanoparticles, rCec-B, and chitosan/Cec-B nanoparticles. One ml of blood was centrifuged at 1000 rpm for 5 min at room temperature followed by washing three times with 1 × PBS (pH7.4) at a ratio of 1:1. The blood was diluted using 1 × PBS at a ratio of 1:10. Twofold dilution (100, 50, 25, 12.5, 6.25, 3.125, 1.6, and 0.8 µg/ml) was added to erythrocytes suspension at a ratio of 4:1, then erythrocytes were incubated for 30 min at 37 °C, followed by centrifugation at 1000 rpm for 3 min at room temperature. The release of hemoglobin from erythrocytes was monitored at 540 nm. The negative control had 1 × PBS instead of a sample, while the positive control received 0.1% Triton X-100 which caused the hemolysis of erythrocytes (Bielawski 1990).

The absorption of the supernatant of erythrocytes lysed in Triton X-100 was defined as being 100% hemolysis.

The hemolysis percentage was calculated using the following equation:

$$\% {\text{ hemolysis}} = \, \left( {{\text{Abs of sample}} - {\text{Abs of }}\left( - \right){\text{ control}}} \right)/{\text{Abs of }}\left( + \right){\text{ control x 1}}00$$

#### Antimicrobial assay—free and encapsulated rCec-B

First, the MIC values of the free peptide were tested on 50 isolates by the broth microdilution test (Weinstein and Lewis 2020). Briefly, pure bacterial cultures from specimens were obtained in brain heart infusion (BHI) agar and incubated at 37 °C for 18 to 20 h. A colony from the pure culture was initially resuspended in sterile water to reach the turbidity of 0.5 McFarland, and the resulting suspension contained approximately 1–4 × 10^8^ colony forming units (CFU)/ml. Using this suspension, a final 1:1000 dilution was performed directly into cation-adjusted Mueller–Hinton broth to obtain a final concentration of 2–7 × 10^5^ CFU/ml. These bacterial inoculums were incubated with different concentrations of tested samples. Serial dilution of free rCec-B, encapsulated rCec-B, and free capsules (100, 50, 25, 12.5, 6.25, 3.125, 1.6, and 0.8 µg/ml) were applied to wells (Romoli et al. 2019). Mixtures of the peptide and inoculums in a final volume of 200 μl were incubated in sterile 96-well polypropylene microplates (Sigma-Aldrich) at 37 °C. A peptide-free control was used for every isolate evaluated. The MIC of free peptide for each isolate was defined as the lowest concentration that inhibited the visible growth of bacteria after incubation for 18 to 20 h. Second, sensitive isolates to free peptide were subjected to the free capsule and encapsulated rCec-B at twofold dilution (100, 50, 25, 12.5, 6.25, 3.125, 1.6, and 0.8 µg/ml). Each isolate and condition was examined in triplicate, and all experiments were run in triplicate (Romoli et al. 2019). The 96-well plate was placed inside a microplate ELISA reader set to a wavelength of 600 nm (Swithenbank et al. 2020).

#### Effect of encapsulated rCec-B on MDR-related genes using qPCR

Studying the effect of encapsulated rCec-B on the expression of genes coding for efflux pump and porin was performed by treating bacterial cells with 50% of obtained MIC. For each isolate, from overnight culture, dilution 1:100 was performed in 96-well culture plates and encapsulated rCec-B was added. The plate was incubated at 37 °C overnight (Zhong et al. 2013). The treated bacterial cells were harvested and RNA extraction was performed using an RNA extraction kit (Biovision, Inc.). Using Novo™ cDNA Kit (Biovision, Inc), SYBR Green master mix (Thermo Fisher Scientific), qPCR was performed. The cycling parameters were: were 95 °C for 15 min followed by 45 cycles of (95 °C denaturation for 20 s, and 60 °C for 1 min (Okasha et al. 2021). The primers sequence (Thermo Fisher Scientific) was designed in this study for each gene, as shown in Table 1. The average of three experiments was used to reflect each marker’s expression ratio. The relative comparative quantitation approach identified the amounts of gene expression.

**Table 1:** Primer sequences for MDR-related gene expression analysis using qPCR

Gene	Sequence	Product length	Reference
*AcrAB*	F: TATTGCGCTGCAGTATCGCT R: GGTATAGCTCTGGGTCACGC	214	This study
*TolC*	F: TTAACAACGTGAACGCGAGC R: GCCACCAGATCCTGTTCGTT	250	This study
*MtdK*	F: TATAGCGCGACCGATATGGC R: ATATAGCCCGCGTTCCACAG	221	This study
*Ompk35*	F: CGAACGCGGCGGAAATTTAT R: CAAAGCTGCCATATTCGCCC	276	This study
*16Srna*	F: TGGAGCATGTGGTTTAATTCGA R: TGCGGGACTTAACCCAACA	159	CDC (2011)

### Statistical analysis

The data were presented as the mean ± SD. The test of significance was performed by GraphPad Prism 8 (San Diego, California, USA) using two-way ANOVA. *p* ∗  < 0.05 was considered as statistically significant difference.

## Results

### Testing integrity of Cec-B peptide using FPLC

**Fig. 1:**
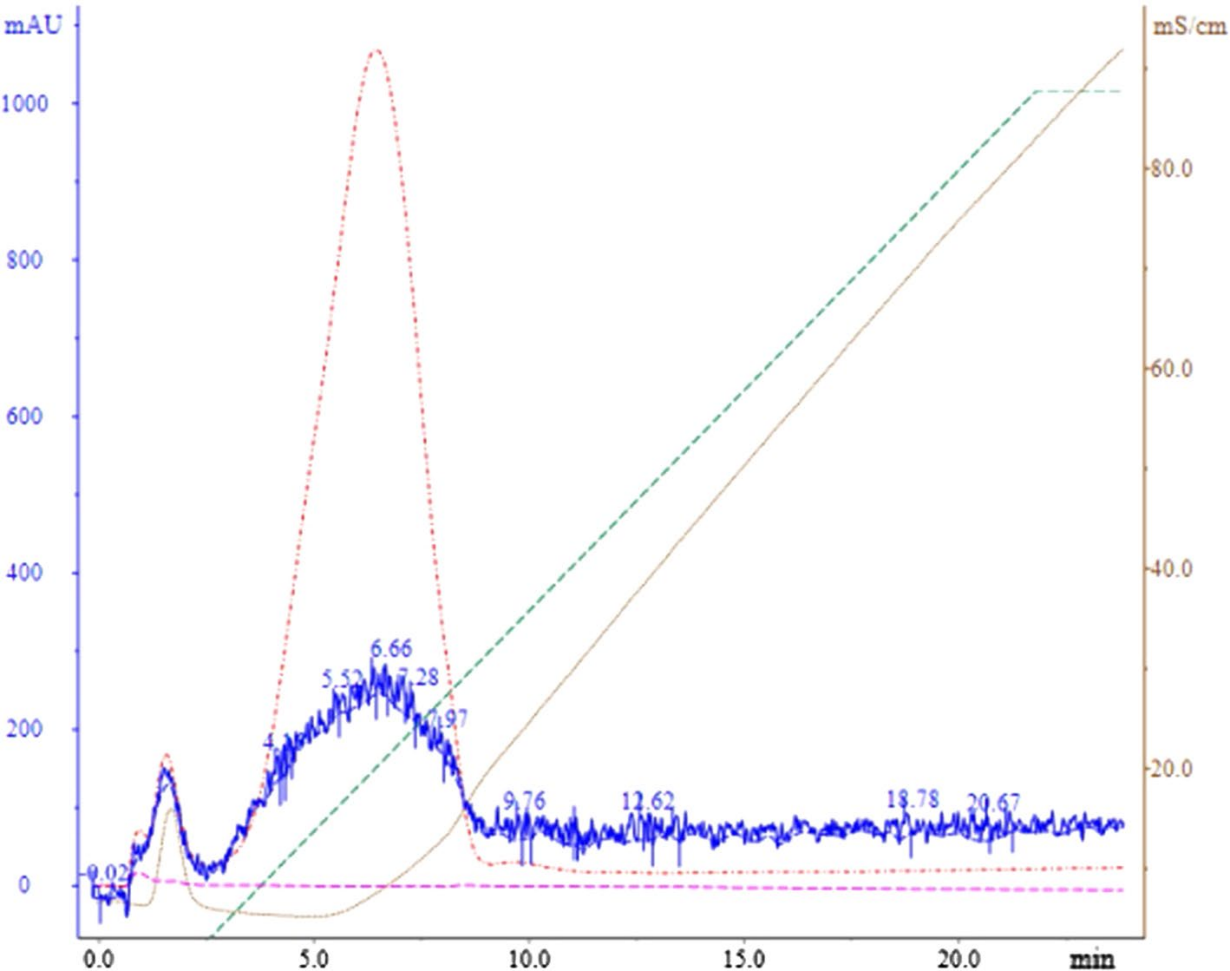
This chromatogram represents the purification of rCec-B using the SP-FF cation exchange column. The X-axis represents time in minutes (min), the primary Y-axis represents the absorbance UV at 190 nm, the red curve represents the absorbance UV at 210 nm, the green curve represents the gradient increasing in salt concentration using elution buffer (0–100% of 1M NaCl), and the secondary Y-axis represents buffer conductivity in Sm/cm

The pH of the buffers employed was 8 since the peptide is cationic in pH ranges below 10.44 according to Cec-B’s pI. As shown in Fig. 1, a sharp peak (UV 210) was detected at retention time from 3 to 9 min in a 12% elution buffer. This peak concludes the stability of rCec-B.

### Efficiency of entrapment

The entrapment efficiency of the chitosan/Cec-B nanoparticles was determined to be 89.2% and the loading capacity to be 39.82%. This percentage reveals the nano-chitosan as a perfect carrier for the delivery of Cec-B.

### Characterization of the chitosan/Cec-B nanoparticles

**Fig. 2:**
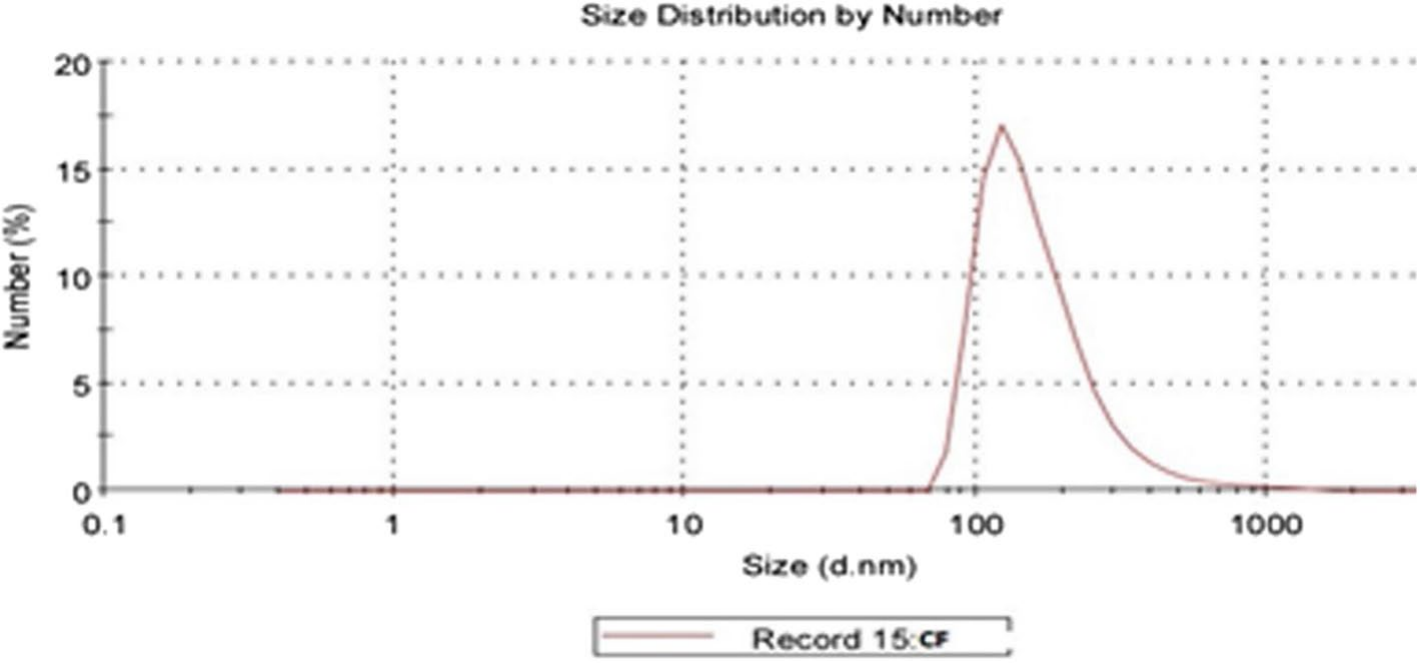
Nano-chitosan (CF)—size: 177.6 nm, PDI: 0.412, and zeta potential: + 29.2 mV

**Fig. 3:**
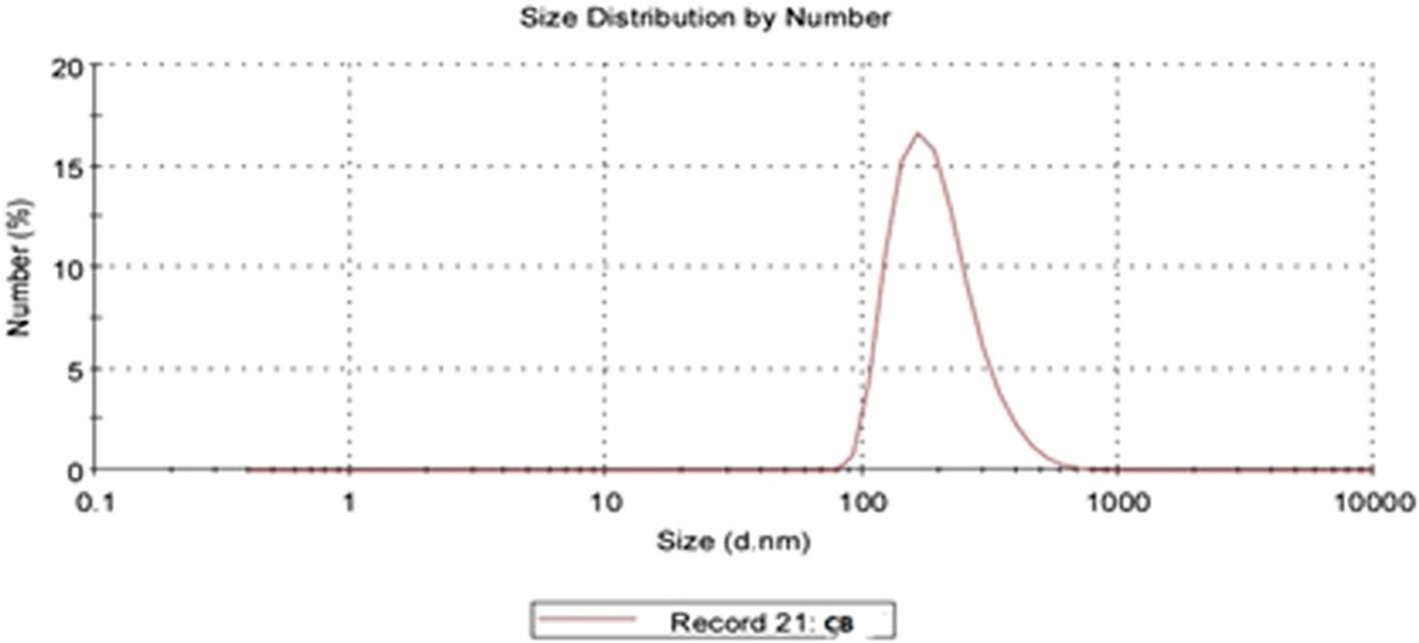
Chitosan/Cec-B nanoparticles (CB)—size = 205.4 nm, PDI: 0.469, and zeta potential =  + 31.3 mV

Upon synthesis and lyophilization, chitosan/Cec-B nanoparticle opalescent suspensions were characterized by dynamic light scattering (DLS) for the hydrodynamic size and the surface zeta potential. The resultant chitosan and chitosan– Cec-B nanoparticles showed a narrow size distribution with hydrodynamic size (177.6 ± 6.92 nm for nano-chitosan and 205.4 ± 10.86 nm for chitosan–Cec-B), polydispersity index of 0.412 ± 0.05 and 0.469 ± 0.04 for chitosan and chitosan–Cec-B nanoparticles, respectively, and a surface charge of 29 − 31 ± 1.3 mV and + 31.3 for chitosan and chitosan–Cec-B nanoparticles, respectively (Fig. 2 and 3), Tables 2, 3

**Table 2:**
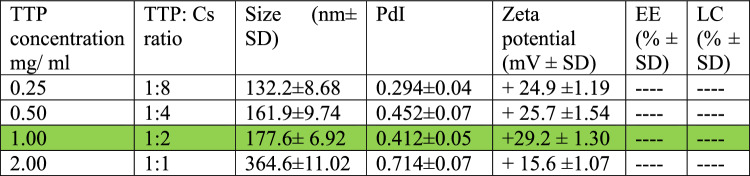
Effect of TTP concentration on size and zeta potential of Cs-NPs

**Table 3:**
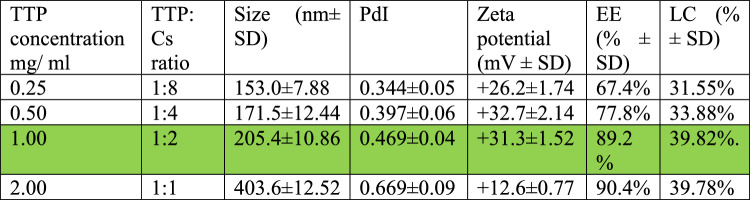
Effect of TTP concentration on size, zeta potential, and encapsulation efficiency of Cs-Cec-B NPs

*TTP* sodium tripolyphosphate, *Cs ratio*: sodium tripolyphosphate: chitosan ratio, *Size*: average diameter distribution, *PdI*: Polydispersity Index, *Zeta potential*: surface charge, *LC*: loading capacity, *EE* encapsulation efficiency

## FTIR analysis

**Fig. 4:**
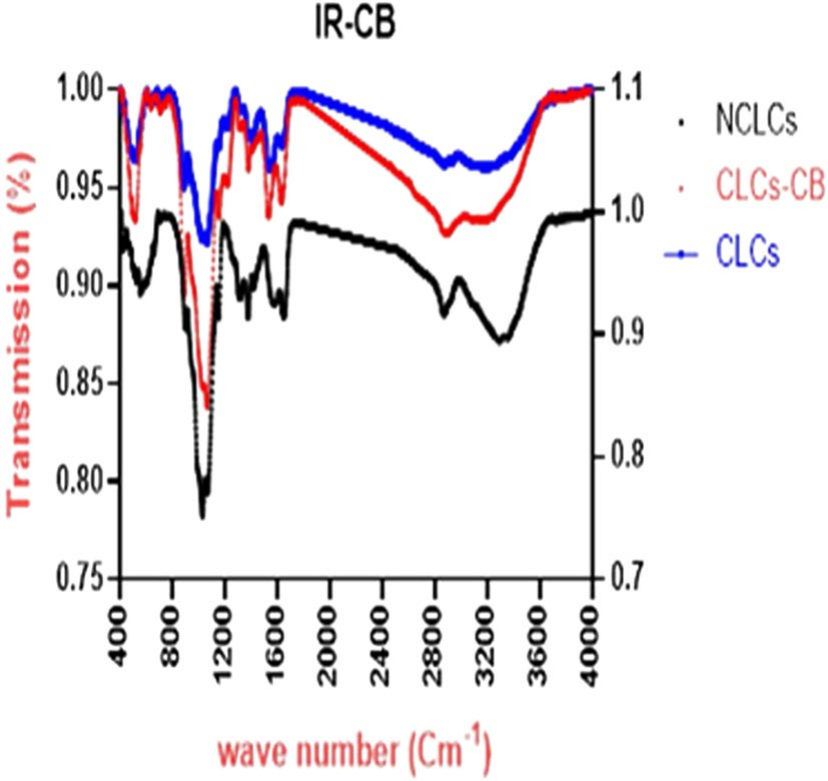
The FTIR spectra of chitosan (NCLCs), chitosan unloaded (CLCs) NPs, and chitosan/Cec-B (CLCs–CB) NPs

The FTIR spectra of chitosan (NCLCs), chitosan unloaded (CLCs) NPs, and chitosan/Cec-B (CLCs-CB) NPs are illustrated in Fig. 4. The appearance, disappearance, or shift of bands reveal the interactions between Cs polymer and TPP cross-linker (69). The broad absorption bands at 3375.02 and 3299.8 cm^−1^ indicate the NH2 and OH groups stretching in Cs. Further, the bands at 2854.3, 1641.22, and 1558.3cm^−1^ resemble the the C–H stretching vibrations, C = O stretching from amide I, as well as N–H bending and C–N stretching from amide II, respectively. Furthermore, the bonds correspond to CH_2_ bending, CH_3_ symmetrical deformation, and primary/secondary OH in-plane bending in the FTIR spectra of Cs appeared at 1413.65, 1378.74, and 1313.37 cm^−1^, respectively. The other prominent bands of non-cross-linked Cs were detected at 1070.37 cm^−1^ (amine C–N stretching) and 1029.86 cm^−1^ (skeletal vibration of C–O stretching). In both the cross-linked Cs and the loaded Cs-NPs, the broad absorption bands at 3375.02 and 3299.8 cm^−1^ indicate that the NH2 and OH groups stretching in Cs were shifted to 3336.45 and 3224.87, the bands at 1641.22, and 1558.3 cm-1 resemble C = O stretching from amide I, as well as N–H bending and C–N stretching from amide II, respectively, also shifted to 1633.51 and 1527.44, another three new bands at 1213, 1157.15, and 858 cm^-1^ were observed. The band at 1415.65 which disappeared the cross-linked Cs was of good intensity in the loaded Cs-NPs. It was also noted that the peak intensity of the C = O stretching from amide I, as well as N–H bending and C–N stretching from amide II, respectively, at 1633.51, and 1527.43 increased and a small peak at 3650.8 cm^−1^ was observed in case of the loaded Cs-NPs.

## Swelling kinetics of chitosan Nps

**Fig. 5:**
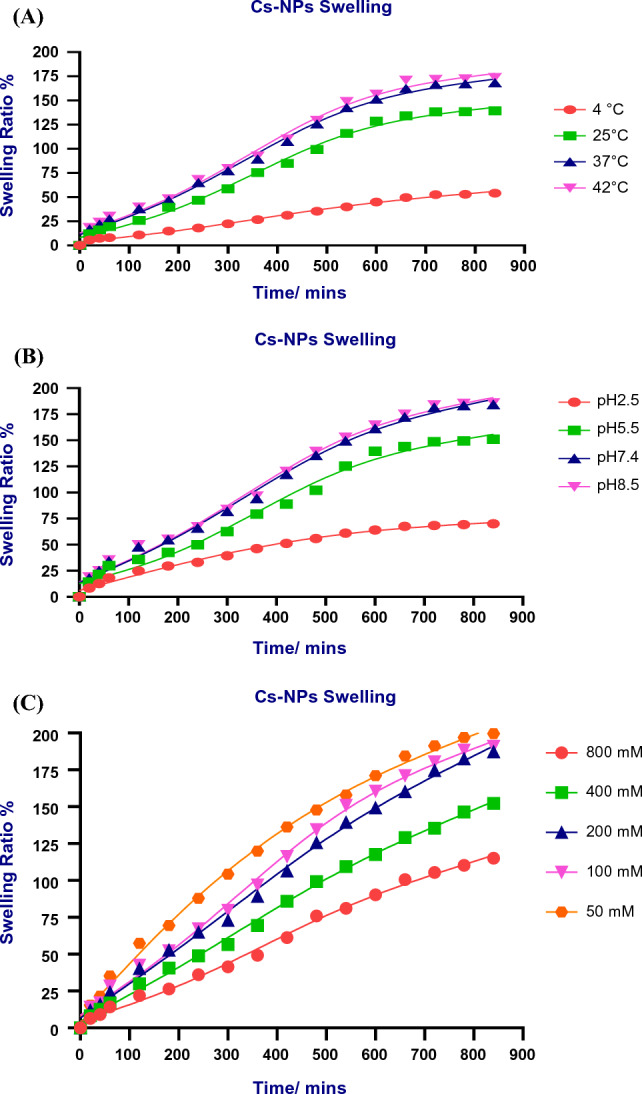
The swelling kinetics of chitosan NPs **A**: effect of temperature, **B** effect of pH, and (**C**) effect of ionic strength

Swelling behaviors of the chitosan NPs at different temperatures, pHs, and ionic strengths are shown in Fig. 5. As the temperature increased from 4 to 42 °C, the polymer swelled faster. This was due to the separation of interpenetrated polymeric chains and the destruction of hydrogen bonding between polymer molecules. At a higher temperature, the chain mobility was increased which facilitated the network expansion. Also, more alkaline pH and higher ionic strengths caused the polymeric NPs to swell faster.

## In vitro drug release of the chitosan–rCec-B nanoparticles

**Fig. 6:**
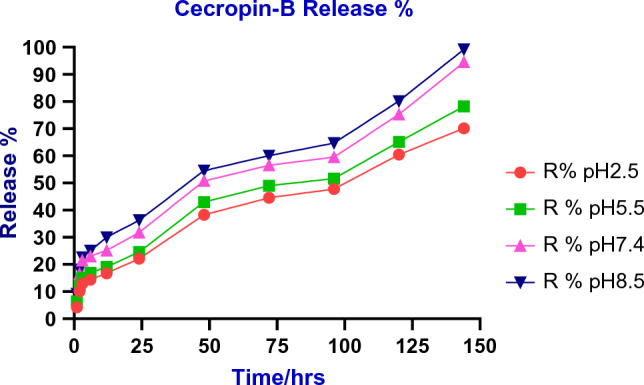
The in vitro release profile of chitosan–rCec-B nanoparticles in PBS at pHs 2.5, 5.5, 7.4, and 8.5

In this study, the in vitro release profile of chitosan–rCec-B nanoparticles in PBS at pH 2.5, 5.5, 7.4, and 8.4 was studied over 7 days. A pH-dependent sustained release of rCec-B was observed, with a maximum release of 99. 22 ± 1.24% at pH 8.5, followed by 94.5 ± 1.67% at pH 7.40, then 78.22 ± 1.85% at pH 5.5 and 70.15 ± 1.54% at pH 2.5 up to 144 h of the study period. (Fig. 6). The exponential pattern demonstrated by the release profile of chitosan–rCec-B nanoparticles indicates that the system is suitable for the sustained release of therapeutics. The amount of rCec-B and chitosan as well as the degree of deacetylation of chitosan plays a vital role in the release rate. The higher the deacetylation of chitosan, the higher the number of amino groups that form ionic interactions with TPP, resulting in the formation of dense particles. This resulted in the lower permeability of the nanoparticle surface and a decrease in release rate.

## Hemolytic activity of the chitosan–rCec-B nanoparticles

**Fig. 7:**
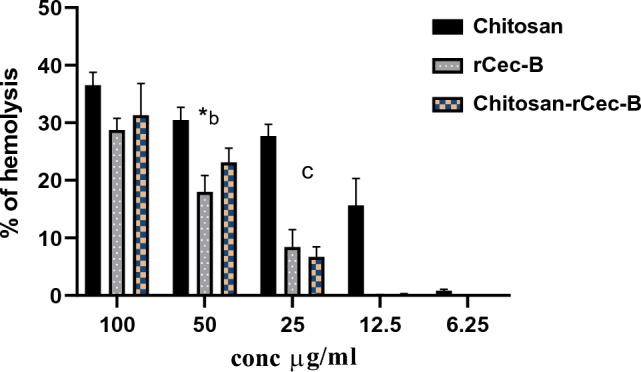
In vitro RBCs toxicity of free chitosan, rCec-B, and encapsulated rCec-B. (*p* value = 0.0002). (*) comparison between rCec-B and chitosan–rCec-B: *p* value < 0.05. (**b**) and (**c**) comparison between chitosan and chitosan–rCec-B: *p* value < 0.01 and *p* value < 0.05, respectively

Results obtained showed low RBCs toxicity (below 50% hemolysis) at a high concentration (100 µg/ml) of a free capsule (hemolysis equals 36.5 ± 2.5%), rCec-B (hemolysis equals 28.7 ± 2%), and encapsulated rCec-B (hemolysis equals 31.3 ± 5.5%) incubated with erythrocytes for 0.5 h at 37 °C compared to triton X-100 as a positive control. At 100 µg/ml: free chitosan showed 36.52 ± 2.3%, rCec-B had 28.75 ± 2%, and encapsulated rCec-B had 31.3 ± 5.5%. However, at 6.25 µg/ml, free chitosan had too low cytotoxicity calculated to be 0.795 ± 0.29%, and zero hemolysis was obtained at the same concentration for both rCec-B and encapsulated rCec-B. Statistical analysis using two-way ANOVA showed high significance between different samples (free capsule, rCec-B, and encapsulated rCec-B) at each tested concentration with *p *value = 0.0002. Statistical significance was found at 50 µg/ml between rCec-B, free capsule, and encapsulated rCec-B. Another significance was obtained between the free capsule and encapsulated rCec-B at the concentration of 25 µg/ml. (Fig. 7).

### Antimicrobial properties of free and encapsulated rCec-B

**Fig. 8:**
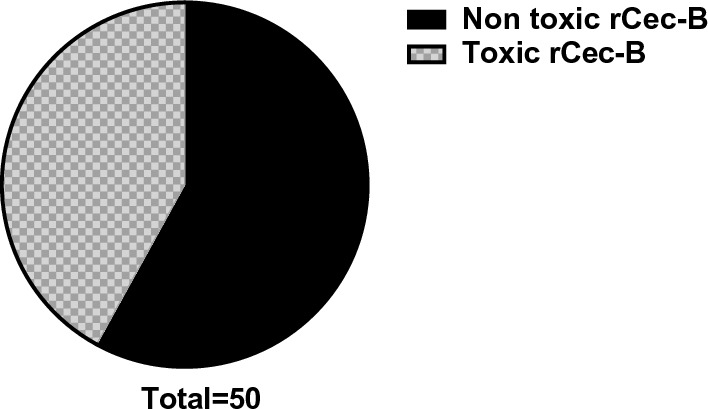
Bactericidal of rCec-B peptide MDR *K. pneumoniae* isolates. The fraction of the total calculation was 0.58 for nontoxic rCec-B and 0.42 for toxic rCec-B

**Fig. 9:**
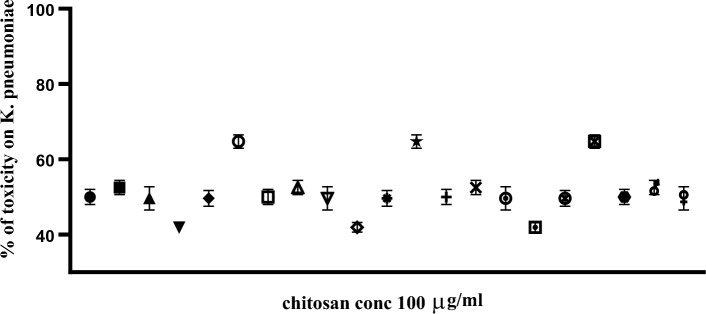
Effect of the chitosan nanocapsule at the highest concentration used (100 µg/ml) on the 21 MDR *K. pneumoniae* isolates. Each dot represents a different isolate. (*p* value < 0.0001)

**Fig. 10:**
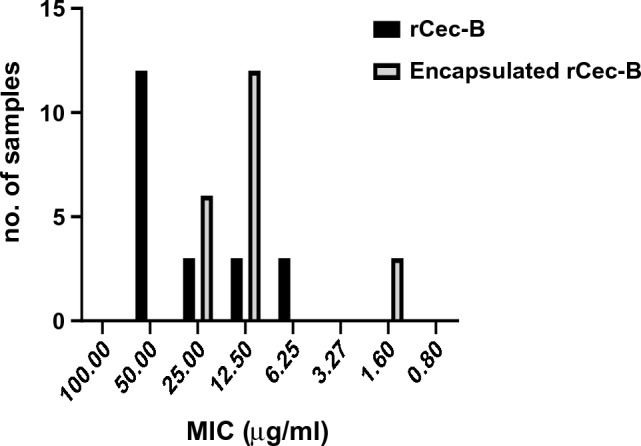
MIC of free and encapsulated rCec-B on the 21 MDR *K. pneumoniae* isolates

**Fig. 11:**
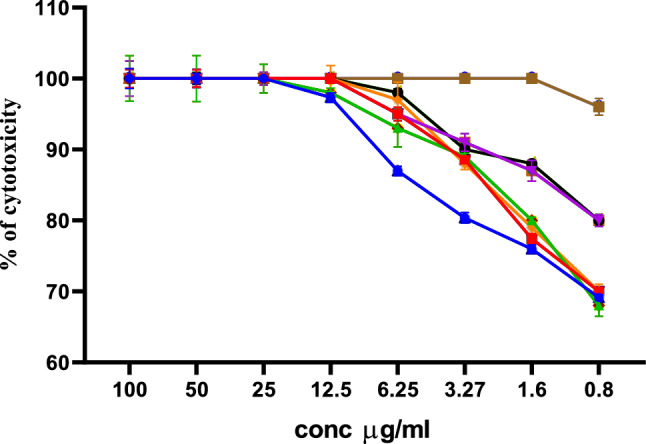
Effect of encapsulated rCec-B on MDR *K. pneumoniae* isolate. (*p* value < 0.0001)

Out of 60 clinical MDR isolates, 50 were MDR-KP. 60% of the isolates were XDR while 40% were MDR. The antimicrobial activity of free peptide was tested first on 50 MDR *K. pneumoniae* isolates and the bactericidal effect was detected on 21 isolates as shown in Fig. 8. Both the free capsule and rCec-B were then tested on these 21 isolates to determine its antibacterial efficacy. Results of the free capsule showed a mild cytotoxic effect on MDR *K. pneumoniae* at the highest concentration, 100 µg/ml, as the highest calculated percent of toxicity on one of MDR *K. pneumoniae* collected isolates was 64.7 ± 1.77% and the lowest percent of toxicity 41.9 ± 1.3% (Fig. 9). The tested free rCec-B had MIC that varied according to *K. pneumoniae* isolates. The highest MIC of free peptide was 50 µg/ml on 12 isolates, MIC was 25 µg/ml on another 3 isolates, MIC was 12.5 µg/ml on another 3 isolates, and MIC was 6.25 µg/ml on another 3 isolates. However, the encapsulated peptide (Chitosan–rCec-B) had the highest MIC at 25 µg/ml on 6 isolates, MIC was 12.5 µg/ml on another 12 isolates, and MIC at 1.6 µg/ml on another 3 isolates. Thus, our results revealed that the MIC of encapsulated rCec-B was higher than the free peptide as represented in Figs. 10 and 11. Two-way ANOVA showed a high significance analysis between the percent of cytotoxicity of encapsulated rCec-B and different tested concentrations, *p *value < 0.0001.

### Effect of encapsulated rCec-B on MDR-related genes using qPCR

**Fig. 12:**
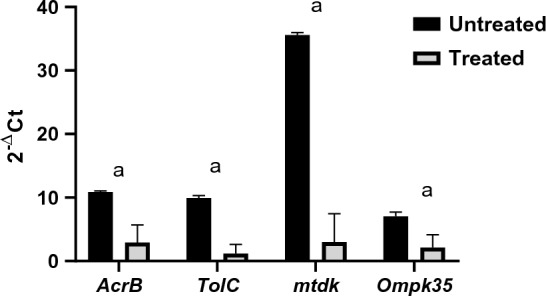
The relative expression levels of MDR-related genes in untreated and treated *K. pneumoniae* isolates. (*p* vlaue < 0.0002), a: *p* vlaue < 0.0001

**Fig. 13:**
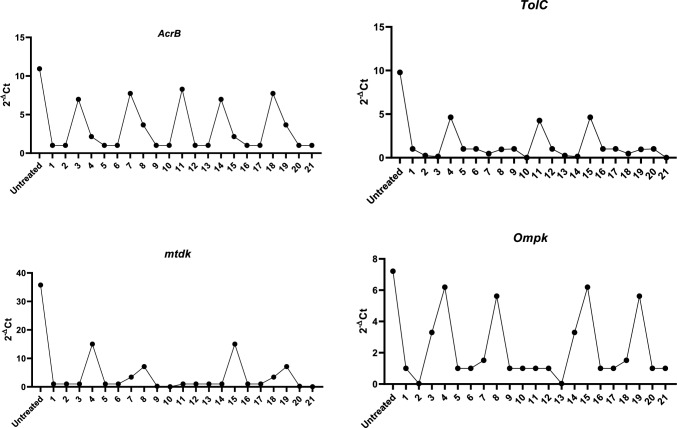
Downregulation of *ArcrB*, *TolC*, *mtdK*, and *Ompk35* genes’ expression as a result of treatment with encapsulated rCec-B

The relative expression of efflux pump and porin coding genes (*ArcrB*, *TolC*, *mtdK*, and *Ompk35*) was detected in treated *K. pneumoniae* bacterial isolates. As shown in Figs. 12 and 13, the expression level of four genes was downregulated after treatment with encapsulated rCec-B. Statistical analysis using two-way ANOVA revealed a high significance between untreated and treated bacteria cells (*p* < 0.0002).

## Discussion

Infections caused by *K. pneumoniae* with multiple antibiotic-resistance genes, associated with several virulence factors, are increasingly reported in hospitalized patients in different parts of the world. These infections have been a global concern, as the therapeutic limitations associated with the pathogenicity of many strains with MDR and XDR phenotypes are related to a large number of morbidity and mortality (Campos et al. 2021; Palmeiro et al. 2019; Samir et al. 2022; Nakamura-Silva et al. 2021; Lam et al. 2021). In response to the rise and escalation of antibiotic-resistant pathogens, antimicrobial peptides (AMPs) have undergone thorough exploration due to their unique characteristics distinct from traditional antibiotics. The current work aims to take advantage of nanoparticle advancement by encapsulating the in-house designed Cecropin-B into chitosan nanoparticles to determine if the incorporation into chitosan NPs might retain the peptide’s antimicrobial activity while reducing its toxicity.

The preparation of the chitosan NPs was performed via the ionic gelation method using appropriate ratios of chtiosan:TPP:rCec-B. The chitosan percentage affects the entrapment efficiency of the recovered particles. A viscous solution (2 mg/ ml Cs) might cause effective dispersion of rCec-B into the polymer matrix, increasing both the efficiency of entrapment and loading capacity. According to Piras et al. (2015), temporin B peptide was encapsulated using chitosan (1 mg/ ml), and only 4.8% temporin B was loaded with an encapsulation efficacy of 75%. Using a concentration of chitosan, 2 mg/ml and a lower peptide concentration (50 µg∕ ml) led to an entrapment efficiency close to 90% in agreement with previous studies stated by Janes and Alonsoand Agnihotri et al. (Janes and Alonso 2003; Agnihotri et al. 2004). In line with the previous study mentioned by Mattu et al., the encapsulation procedure at pH 5.5 resulted in higher entrapment efficiency (Mattu et al. 2013).

Among the numerous obstacles in the development of antimicrobial peptides (AMPs) for clinical use is their markedly diminished antimicrobial effectiveness attributed to low stability in specific environments (Lei et al. 2019). The pH and thermal stability of AMPs are essential for their preparation, processing, and storage (Wong et al. 2019). At the same time, the stability of AMPs in serum and under physiological salt conditions is also crucial when administrating AMPs in vivo (Mishra et al. 2019).

In agreement with previous reports, the dynamic light scattering analysis of the current study showed us that the particle size and zeta potential of formulated chitosan NPs increased significantly when the concentration of chitosan was increased. The zeta potential on the surface of nanoparticles indicates the particles are stable. Nanoparticles possessing higher negative or positive zeta potentials will tend to repel each other and not form aggregates (Bilal et al. 2019; Cinteza et al. 2018).

According to the pI of rCec-B, the peptide is cationic in pH below 10.44. During the formulation of chitosan–rCec-B NPs, the pH was maintained at 5.5 to facilitate the conjugation of chitosan with rCec-B peptide and produce a carrier with enhanced ability to facilitate cellular uptake. Acidification of chitosan in addition to protonation of these amino groups makes chitosan positively charged (Wang et al. 2006). Chitosan’s -NH ^+2^ groups are changed to the soluble protonated NH ^3+^ form when the pH of the solution falls below its pKa value of 6.3, making it soluble in acidic aqueous solutions. This soluble protonated NH 3 + form, in turn, reacts with the negatively charged phosphate ions of TTP and results in stable nanoparticles. The characteristic absorption peaks at 1213, 1157.15, and 858 cm^−1^ indicate the successful interaction between chitosan (-NH_3_^+^ groups) and TPP (P_3_O_10_^5–^ groups which dissociate in water to give both hydroxyl -OH^−^ and phosphoric ions) and the P = O groups were also detected in the absorption bands at wave numbers of 1157.15 and 858 cm^−1^ (Shah et al. 2016) whereas the peaks at 3650.8, 1633.51, 1527.43, and 1415.65 cm^−1^ resemble the loading of the peptide on the chitosan NPs and the bands of amide I and amide II dramatically shifted to 1633.51 and 1527.43 cm^−1^. These results are in agreement with the previous report which studied the antimicrobial efficacy of chitosan-encapsulated Cecropin-A (1–7)–melittin-cell-penetrating peptide against multi-drug-resistant *Salmonella enteritidis* (Wu et al. 2017). Similar results were observed by Xu et al. and Devika et al., in their study of the formation of chitosan NPs and chitosan film treated with phosphate (Xu and Du 2003; Bhumkar and Pokharkar 2006).

The temperature-dependent equilibrium swelling behavior of the chitosan NPs in deionized water (pH 7) at a temperature range from 4 to 42 °C was observed. As the temperature of the NPs in the swelling state increased, the swelling ratio of the NPs samples increased. All particles exhibited a temperature-responsive swelling behavior due to the association/dissociation of hydrogen bonding between the amino groups within the chitosan chains. Less swelling was observed with increasing temperature because of increased solubility. The obtained results were in accordance with many conducted studies in which they studied the swelling properties of chitosan hydrogels compared to other pH-sensitive polymeric hydrogels (Rohindra et al. 2004; Vishal Gupta and Shivakumar 2012).

Because the rCec-B release from the chitosan NPs was accomplished via a variety of mechanisms, including diffusion and erosion, the pH-dependent release profile revealed that the maximum release occurred at pH 8.5, whereas the lower release was at low pH 2.50. This lower release is due to the protonated chtiosan’s amino group, which protects rCec-B from acidic pH. In this study, the highest release of rCec-B within 24 hours was ascribed to the diffusion of rCec-B that was loosely entrapped. Additionally, a continuous, gradual release was observed, which was attributed to the relaxation of chitosan and sodium tripolyphosphate (STPP) ionic crosslinking in both physiological pH (7.40) and alkaline pH. These results are in accordance with the previously published reports (Wu et al. 2017).

The release of drugs from the chitosan NPs is affected by the hydrophilicity of chitosan and the pH of the swelling solution. Due to the hydrophilicity of chitosan, chitosan NPs exhibit a pH-dependent drug and controlled drug release system. The larger amount of encapsulated rCec-B leads to a higher diffusion rate due to the formation of a concentration gradient between the chitosan and buffer matrix (Wang et al. 2006). Since the rCec-B encapsulated was a small amount and chitosan–rCec-B NPs showed 89.2% entrapment efficiency, the release rate was slow. That is because the chitosan used in the study is of low concentration (2 mg/ml) and high degree of deacetylation.

The results of the release study agreed with the previous study reported by Jayathilaka et al. (2022). The maximum cumulative release was 88.26% at 96 h using low-molecular-weight chitosan. Another study reported a maximum BSA cumulative release of 72.52% at 350 h using low-molecular-weight chitosan (60). Whereas, the release kinetics of the peptide from temporin-B-loaded chitosan (medium molecular weight, Mw108 kDa (Mw/Mn2.4), deacetylation degree ∼92%) NPs was studied in SPB pH 7.4 for 15 days with the maximum cumulative release of about 18. %. After the first equilibration time (lag time), the system displayed a progressive linear release.

The antimicrobial-resistant pattern for *K. pneumoniae* isolates showed that most of the isolates were XDR (60%) and 40% were MDR. These results are comparable to a study by Al-Baz et al. who reported that most of the isolates were XDR (60.6%) and 30.3% were MDR (Lam et al. 2021). A study in Minya, Egypt by Hassuna et al. reported an alarming occurrence of XDR *K. pneumoniae* with an incidence of 83.3% (Hassuna et al. 2020). Different figures were shown in a study done by Aamir et al. who reported that 47.2% were MDR and 36.1% were XDR (Aamir et al. 2021). Many factors may contribute to the spread of MDR and XDR isolates such as limited adherence to infection control protocols and unnecessary use of antimicrobials.

Based on our findings, rCec-B showed antibacterial activity against *K. pneumoniae* with MIC values between 50 – 6.5 μg/mL. The MICs in this study were comparable to those reported for other cationic peptides Ocampo-Ibáñez et al. reported that cecropin D-derived showed bactericidal effect with MIC values between 32 and > 256 μg/mL (Ocampo-Ibáñez et al. 2020); However, slightly higher in comparison with those reported for the Cecropin-A–melittin hybrid peptide (Geitani et al. 2019).

In our study, the free capsule showed a mild cytotoxic effect on MDR *K. pneumoniae* at the highest concentration. The results of the present study were in line with the study performed by Hassan et al., 2021, where the antimicrobial activity of different concentrations of chitosan nanoparticles against five isolates of MDR *K. pneumonia*e showed no antibacterial activity of all chitosan nanoparticles concentrations against the bacteria (Hassan et al. 2021). Another study also showed a disparity with our data, where Zhang et al. (Zhang et al. 2020) showed that diluted chitosan-loaded essential oil has increased antibacterial activity against tested *K. pneumoniae* after overnight incubation (Zhang et al. 2020).

Our results revealed that the MIC of chitosan–rCec-B NPs was higher than the free rCec-B as represented. This was in line with the study of Herdiana et al. where the antibacterial activities of Wasp chitosan-based NPs (WCSNPs) against positive *K. pneumoniae*, *E coli*, and *P. aeruginosa* were investigated (Herdiana et al. 2022). The results indicated that the percentage of growth inhibition of the synthesized nanomaterials can credibly help fight the growth of ESBL- and carbapenem-resistant *K. pneumoniae*, *E. coli*, and *P. aeruginosa* upon increasing concentration. In this study, the distribution of efflux-resistant genes was significantly high in the studied isolates (100%). Moreover, a substantial expression of the AcrAB gene; responsible for the multidrug efflux pump system, and the tolC gene; encoding the transport channel protein, was identified. This finding aligns with the observations made by Wasfi et al. Surprisingly, OmpK35 porins were identified in all isolates, and this presence was elucidated by the existence of point mutations or disruption the protein coding sequence, (Wasfi et al. 2016). On the other hand, Ompk35 also plays a role in *K. pneumoniae* virulence and infection. Statistical analysis also showed a significant strong positive correlation between antibiotic-resistance patterns and the presence of resistance genes. Thereby, these genes may be implicated in the resistance of *K. pneumoniae* to antibiotics. The expression level of four genes was downregulated after treatment with encapsulated rCec-B. In line with our results, it was shown that AgNPs could suppress antimicrobial resistance through the activation of the efflux pump system in *K. pneumoniae* (Dolatabadi et al. 2021). Another recent study investigating the underlying antimicrobial mechanism of lysozyme-coated AgNPs against AMR *K. pneumoniae* (MGH78578, ATCC® 700,721) using transcriptomics analysis identified oxidative stress and a triclosan-like antibacterial mechanism (Alotaibi et al. 2022).

## Conclusion

In conclusion, the results of this study demonstrated the activity of CAMP-Cec-B with MDR *K. pneumoniae*. This peptide exhibited antibacterial activity against MDR strains of *K pneumoniae* with down-regulation of porin and efflux pump encoding genes. In this context, rCec-B emerges as a prospective alternative pharmaceutical agent to traditional antibiotics for managing multidrug-resistant bacteria linked to severe infectious diseases.

## Data Availability

All data generated or analyzed during this study are included in this published article.
